# Electrophysiological correlates of the interplay between low-level visual features and emotional content during word reading

**DOI:** 10.1038/s41598-018-30701-5

**Published:** 2018-08-15

**Authors:** Sebastian Schindler, Antonio Schettino, Gilles Pourtois

**Affiliations:** 1 Department of Experimental-Clinical and Health Psychology, Ghent University, Ghent, Belgium; 2 Department of Psychology, University of Bielefeld, Bielefeld, Germany; 3 Institute of Medical Psychology and Systems Neuroscience, University of Muenster, Muenster, Germany; 4 Institute for Globally Distributed Open Research and Education (IGDORE), Ubud, Indonesia

## Abstract

Processing affectively charged visual stimuli typically results in increased amplitude of specific event-related potential (ERP) components. Low-level features similarly modulate electrophysiological responses, with amplitude changes proportional to variations in stimulus size and contrast. However, it remains unclear whether emotion-related amplifications during visual word processing are necessarily intertwined with changes in specific low-level features or, instead, may act independently. In this pre-registered electrophysiological study, we varied font size and contrast of neutral and negative words while participants were monitoring their semantic content. We examined ERP responses associated with early sensory and attentional processes as well as later stages of stimulus processing. Results showed amplitude modulations by low-level visual features early on following stimulus onset – i.e., P1 and N1 components –, while the LPP was independently modulated by these visual features. Independent effects of size and emotion were observed only at the level of the EPN. Here, larger EPN amplitudes for negative were observed only for small high contrast and large low contrast words. These results suggest that early increase in sensory processing at the EPN level for negative words is not automatic, but bound to specific combinations of low-level features, occurring presumably via attentional control processes.

## Introduction

Attention mechanisms enable the parsimonious and efficient allocation of cognitive resources by selecting stimuli and features that are goal-relevant or salient^1,2^. Among the possible ways in which the brain may tag something as relevant, there are bottom-up factors (e.g., abrupt changes in luminance), top-down factors (e.g., a task must be accomplished), and biological significance (e.g., social, motivational, and emotional meaning).

Within the visual domain, bottom-up perceptual relevance has typically been examined by manipulating low-level properties of the stimuli. For example, electrophysiological studies have shown that changes in visual contrast or stimulus size modulate the amplitude of P1 and N1 event-related potential (ERP) components, which are thought to reflect early cognitive processes associated with stimulus detection and discrimination, respectively^3–5^. Manipulating visual contrast – e.g., by showing dark compared to bright stimuli on a uniform background – typically elicits larger amplitude and delayed peak latency of the P1 and N1 components^6–8^. Similarly, size manipulation was found to affect both P1 and N1, with larger size leading to increased amplitude^9^.

Attention can also voluntarily be allocated to features that are relevant for the task at hand, via spatial cues^10,11^ or task instructions^12,13^. Findings typically show that top-down attention manipulations to visual stimuli increase the amplitude of P1 and N1 components^3,4,10,14^. These early ERP components are less affected by secondary or preceding tasks (e.g., attentional blink)^15,16^ or evaluative processes (e.g., classifying or focusing on emotion)^17–20^.

A third source of perceptual relevance is emotional content^21,22^. Processing affectively charged visual stimuli – e.g., words^23^, faces^24^, naturalistic scenes^20^, or videos^25^ –, compared to their neutral controls, typically results in increased amplitude of specific ERP components, namely the Early Posterior Negativity (EPN) and the Late Positive Potential (LPP). The EPN arises at about 200 ms following stimulus onset and is related to early attentional selection^26,27^. The LPP occurs from about 400 ms after stimulus onset and reflects more elaborative and controlled processes, which are related to sustained attention, stimulus evaluation, affective labeling, and episodic memory formation^28,29^.

Importantly, bottom-up, top-down, and biological relevance do not act in isolation, but are interconnected^22,30^. Previous work has investigated how bottom-up stimulus features – e.g., spatial frequency^31^, color^32^, size^33^, picture complexity^34^, and brightness^35^ – may modulate behavioral and electrophysiological responses to emotional scenes. A parametric increase in stimulus size of emotional pictures may lead to growing subjective emotional arousal as well as selective amplification of the EPN^33,36^. Moreover, interactive effects of attention allocation towards emotional (i.e., erotic) material and processing of picture brightness have been reported at the level of the N1 component, whereas the EPN and LPP were reliably modulated by emotional content only^35^ – in contrast to the reported interactions of size and emotion at the EPN level. Higher visibility of emotional scenes by concurrent frequency filtering and size manipulation seems to have similarly increasing attentional effects when measuring reaction times and skin conductance response^37,38^.

Besides pictorial stimuli, many studies have used emotional and neutral words to investigate how the human brain processes emotional semantic information^39,40^. An advantage of using words over complex scenes is that luminance, spatial frequency content, and other perceptual statistical regularities can more easily be controlled (however, other non-emotional stimulus features – e.g., frequency^41–44^, length^43^, age of acquisition^42^ – influence recognition speed and accuracy, and must therefore be carefully matched across emotion classes). In general, emotional (compared to neutral) words are categorized more quickly and efficiently^26,45^, and concurrently elicit larger EPN and LPP amplitudes^46–49^. Emotion-dependent amplitude increases of early ERP components – i.e., P1 and N1 – are still under debate, due to mixed findings reported in the existing literature^39^.

Recent studies have explored whether changes in low-level visual properties can also modulate word processing. Bayer and colleagues^50^ presented positive, negative, and neutral words in either small or large font size, while requiring participants to perform an orthogonal *1*-back task to ensure semantic processing of all stimuli. Large words elicited increased P1 and decreased N1 amplitudes, but no emotion-dependent modulations. Statistically significant interactions between font size and emotional content were observed in the late portion of the EPN, with more negative amplitude for emotional than neutral words further amplified when font size was large. The authors interpreted these results as reflecting early interactions of stimulus-driven, bottom-up properties with emotional content, in addition to the aforementioned top-down interactions with emotion at late stages. These authors further argued that sensory facilitation for motivationally relevant stimuli, initially thought to occur only for pictorial stimuli, might be generalized and extended to written words due to the high social relevance of language.

Despite these recent advancements, it is still unclear whether: (i) amplitude modulations of the aforementioned ERP components are limited to font size or may also be generalized to other visual features; (ii) emotion-related ERP amplitude amplification during visual word processing is related to low-level-feature changes or, instead, occurs independently from them. In the current study, forty participants were presented with unpleasant and neutral Dutch words shown in a large or small font size and in high or low contrast relative to a homogenous background. The task was to press the spacebar as soon as a word referring to a color would appear on screen. Hence, semantic processing of the words was required throughout the experiment. Using model comparison via Bayes factors, we examined ERP responses associated with early sensory and attentional processes as well as early lexical and later stages of processing. Bayes factors allow to quantify the evidence in favor of one model relative to another, e.g., a model that assumes medium-sized differences between conditions as opposed to a model that assumes no differences (for details, see *Section 4.6*).

Based on published findings, we expected to replicate the interaction between size and emotional content found at the level of the EPN^50^, showing increased emotion effects for larger words. Furthermore, if sensory facilitation can truly be generalized to symbolic material^39,40,51^, we should be able to observe not only size- but also contrast-dependent effects, specifically N1 amplitude modulations similar to what has been reported when using naturalistic scenes^35^. We additionally speculated that emotion might enhance visual processing especially for stimuli that are harder to discriminate (e.g., small, low-contrast words). We also addressed whether additive or interactive models best explained the ERP data, in contrast to published findings testing only main effects or interactions.

These theoretical predictions, together with a detailed description of the sampling criteria and analysis pipeline, were pre-registered on the Open Science Framework (https://osf.io/uf9gh/). Pre-registration effectively minimizes hindsight or confirmatory biases, since the research questions and analysis plans are defined *before* observing the outcome^52^.

## Results

**Figure 1:**
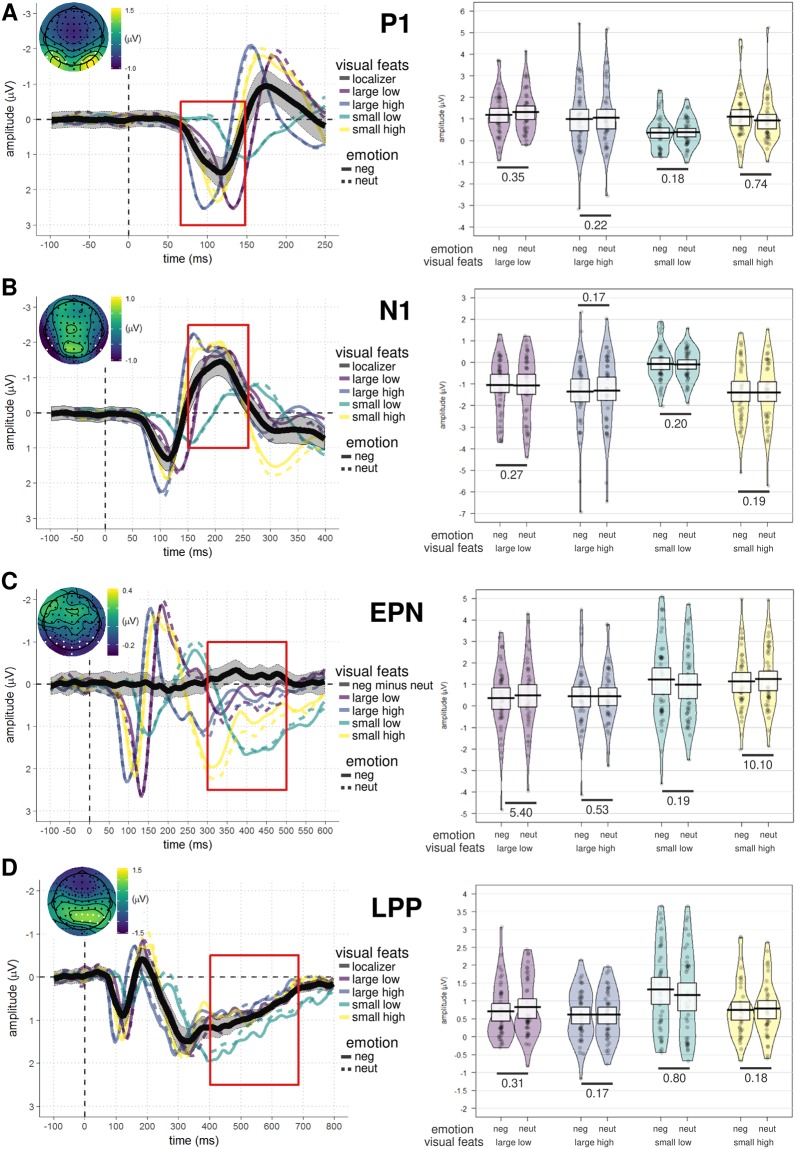
ERP waveforms, topographies, and amplitude values of each ERP component. The panels are divided according to component: (**A**) P1; (**B**) N1; (**C**) EPN; (**D**) LPP. Left panels show the grand average ERP waveforms, separately for each condition (see legends for the respective colors) and averaged across all conditions (black line, shaded area representing 95% confidence intervals). The signal was extracted from electrodes with signal robustly different from noise (highlighted in white in the topography; see *Section 4.5* for details). Of note, the EPN was not extracted by averaging all conditions, but by computing the difference between negative and neutral conditions (irrespective of font size and contrast; see *Section 4.5* for the rationale behind this choice). Right panels show the amplitude values of the respective component for each participant (gray dots) and experimental condition. Mean amplitude values are marked by horizontal black lines and 95% Bayesian highest density interval (HDI) are displayed as white boxes. Numbers represent, for each visual feature combination, the Bayes factors (*BF*_*10*_) in favor of the alternative model – hypothesizing differences between emotion conditions (prior on effect sizes with location *δ* = 0 and scaling factor *r* = 0.707) – versus the null model (difference between emotion conditions *δ* = 0). For details, see *Section 4.6* and Table 3. Abbreviations: *large low*: large size, low contrast; *large high*: large size, high contrast; *small low*: small size, low contrast; *small high*: small size, high contrast; *neg*: negative; *neut*: neutral; *localizer*: average of all conditions; *neg minus neut*: difference between negative and neutral conditions (averaged across font size and contrast).

The P1, N1, EPN, and LPP components were identified in the grand-averaged ERP signal using the mass univariate analysis approach described in *Section 4.5*. Average amplitude values for each component and condition can be found in Table 1. The left panel of Fig. 1 displays the waveforms and topographies for each component separately.

**Table 1:** Means and standard deviations (in parenthesis) of amplitude values (in *µV*) of the P1, N1, EPN, and LPP components.

component	large				small			
	high		low		high		low	
	negative	neutral	negative	neutral	negative	neutral	negative	neutral
P1	1.00 (3.82)	1.06 (3.76)	1.21 (3.76)	1.34 (3.76)	1.09 (3.81)	0.92 (3.73)	0.34 (3.83)	0.38 (3.86)
N1	−1.36 (3.67)	−1.38 (3.69)	−1.03 (3.69)	−1.13 (3.76)	−1.39 (3.67)	−1.45 (3.71)	−0.03 (3.73)	−0.09 (3.86)
EPN	0.33 (3.81)	0.53 (3.75)	0.27 (3.93)	0.58 (3.88)	1.04 (3.86)	1.37 (3.84)	1.15 (4.01)	1.09 (4.11)
LPP	0.61 (3.04)	0.60 (2.95)	0.71 (3.03)	0.81 (3.04)	0.76 (3.09)	0.79 (3.09)	1.35 (3.31)	1.17 (3.36)

*Note*. Values are extracted from electrode clusters identified with the mass univariate procedure (see *Section 4.5* for details). Factors are: *emotion*, negative or neutral; *size*, large or small; *contrast*, high or low.

Bayes factors are reported on the *log* scale. For ease of readability, only results obtained with JZS priors with location *δ* = 0 and scaling factor *r* = 0.707 are reported in the main text. Results obtained with other scaling factors can be found in the respective tables.

### P1

Mean amplitude values of the P1 component were best explained by the *size* × *contrast* × *emotion* interaction model, with a BF_10_ of *e*^514.57^ = 2.98 × 10^223^ relative to the *null* model. In other words, a model including all three factors and their interactions explained the observed data 2.98 × 10^223^ times better than a null model, i.e., not including any independent variables. The full model was also *e*^514.57-490.49^ = *e*^24.08^ = 2.87 × 10^10^ times better than the second-best model assuming additive effects of size, contrast, and emotion (Table 2). However, follow-up contrasts showed no reliable amplitude differences as a function of emotional content. Specifically, evidence leaned in favor of the *null* model when assessing emotion differences of words presented in small size and low contrast (BF_10_ = *e*^−1.72^ = 0.18), large size and high contrast (BF_10_ = 0.21), large size and low contrast (BF_10_ = 0.35), or small size and high contrast (BF_10_ = 0.73; inconclusive) (Table 3).

**Table 2:** Model comparisons, separately for each ERP component.

component	Model	*r* = *0.5*		*r* = *0.707*		*r* = *1*	
		BF_10_	% pe	BF_10_	% pe	BF_10_	% pe
P1	**size** × **cont** × **emo**	**517.25**	**±26.10**	**514.57**	**±21.76**	**512.82**	**±38.59**
	size + cont + emo	491.61	±12.25	490.49	±11.28	489.70	±12.94
	size + emo	488.04	±10.98	487.25	±13.15	486.48	±15.14
	size × emo	485.31	±13.64	484.26	±14.00	483.27	±14.79
	cont + emo	455.18	±10.52	454.54	±10.86	453.44	±11.01
	cont × emo	451.90	±11.86	451.12	±15.34	450.06	±17.30
N1	**size** × **contr** × **emo**	**1,136.06**	**±17.41**	**1,133.45**	**±7.68**	**1,130.97**	**±7.70**
	size + cont + emo	1,095.33	±3.84	1,094.31	±3.89	1,093.33	±3.86
	cont + emo	1,054.64	±3.28	1,053.99	±3.47	1,053.30	±3.32
	cont × emo	1,050.90	±4.21	1,049.97	±4.21	1,048.95	±4.57
	size + emo	968.70	±3.56	968.00	±3.43	967.29	±3.46
	size × emo	964.94	±4.22	963.88	±4.34	962.80	±4.43
EPN	**size + emo**	**1,326.56**	**±2.33**	**1,325.85**	**±2.36**	**1,325.17**	**±2.40**
	size × emo	1,323.40	±3.04	1,322.38	±3.07	1,321.31	±3.03
	size + cont + emo	1,322.78	±2.97	1,321.78	±3.01	1,320.75	±2.96
	size × cont × emo	1,312.82	±5.84	1,310.22	±6.12	1,307.83	±7.52
	contr + emo	1,232.28	±2.47	1,231.57	±2.40	1,230.90	±2.62
	cont × emo	1,229.30	±3.27	1,228.28	±2.96	1,227.24	±3.13
LPP	**size + cont + emo**	**520.75**	**±11.58**	**519.6**	**±14.21**	**518.57**	**±12.27**
	size × cont × emo	515.67	±15.47	513.49	±15.62	511.30	±26.89
	size + emo	496.26	±10.65	495.59	±11.47	493.47	±0.00
	cont + emo	494.06	±9.85	493.60	±11.74	492.97	±10.90
	size × emo	493.14	±12.55	492.07	±10.01	491.39	±12.85
	cont × emo	490.48	±12.07	489.49	±11.99	488.63	±15.22

*Note*. Bayes factors (BF_10_, on *log* scale) and percentage of proportional errors (% pe) for each model relative to the null, obtained by using JZS priors with different scaling factors (see *Section 4.6* for details). The model best explaining the data for each component is highlighted in bold. Factors are: *emotion* (emo), negative or neutral; *size*, large or small; *contrast* (cont), high or low.

**Table 3:** Post-hoc comparisons, separately for each ERP component.

component	post hoc comparison	*r* = *0.5*		*r* = *0.707*		*r* = *1*	
		BF_10_	% pe	BF_10_	% pe	BF_10_	% pe
P1	large size, high contrast, negative vs. neutral	1.24	±0.00	−1.54	±0.00	−1.85	±0.00
	small size, high contrast, negative vs. neutral	−0.08	±0.00	−0.31	±0.00	−0.58	± 0.00
	large size, low contrast, negative vs. neutral	−0.80	±0.00	−1.06	± 0.00	−1.36	±0.00
	small size, low contrast, negative vs. neutral	−1.41	±0.00	−1.72	±0.00	−2.04	±0.00
N1	large size, high contrast, negative vs. neutral	−1.44	±0.00	1.75	±0.00	−2.08	±0.00
	small size, high contrast, negative vs. neutral	−1.34	±0.00	−1.64	±0.00	−1.96	±0.00
	large size, low contrast, negative vs. neutral	−1.04	±0.00	−1.33	±0.00	−1.63	±0.00
	small size, low contrast, negative vs. neutral	−1.31	±0.00	−1.60	±0.00	−1.92	±0.00
EPN	large size, high contrast, negative vs. neutral	−0.40	±0.00	−0.64	±0.00	−0.92	±0.00
	**small size, high contrast, negative vs. neutral**	**2.41**	±**0.00**	**2.31**	±**0.00**	**2.13**	** ± 0.00**
	**large size, low contrast, negative vs. neutral**	**1.81**	±**0.00**	**1.69**	±**0.00**	**1.49**	±**0.00**
	small size, low contrast, negative vs. neutral	−1.37	±0.00	−1.67	±0.00	−1.99	±0.00
LPP	large size, high contrast, negative vs. neutral	−1.46	±0.00	−1.77	±0.00	−2.09	±0.00
	small size, high contrast, negative vs. neutral	−1.39	±0.00	−1.69	±0.00	−2.02	±0.00
	large size, low contrast, negative vs. neutral	−0.91	±0.00	−1.18	±0.00	−1.48	±0.00
	small size, low contrast, negative vs. neutral	−0.01	±0.00	−0.23	±0.00	−0.49	±0.00

*Note*. Bayes factors (BF_10_, on *log* scale) and percentage of proportional errors (% pe) for each model assuming pairwise differences between conditions relative to the null model (details are provided in *Section 4.6*). BF_10_ above zero indicates better fitting for the alternative compared to the null model. Post-hoc comparisons in favor of a difference are highlighted in bold.

In a following step, we sought to assess the separate contribution of size, contrast, and emotion by including all possible models, i.e., not only the theoretically relevant ones that always included emotion. We started with the *full* model and progressively tested all models that could be created by removing one interaction or main effect one at a time (“*top-down analysis*”; see http://bayesfactorpcl.r-forge.r-project.org/#fixed). This procedure revealed that omitting the factor *emotion* from the full model improved fitting by 82.27 times. Removing the interactions *size* × *contrast* × *emotion*, *contrast* × *emotion*, and *size* × *emotion* also improved fitting by 35.52, 24.29, and 21.54 times, respectively. Thus, *emotion* did not seem to have any explanatory power; instead, it penalized the models in which it was included. Conversely, omitting the factor *contrast* or the *contrast* × *size* interaction lowered the explanatory value of the resulting model by 1/*e*^−3.75^ = 42.51 and 3.57 × 10^14^ times, respectively. Finally, removing the factor *size* was maximally detrimental, as it would lower the explanatory value of the resulting model by 9.79 × 10^15^ times (see Table 4).

**Table 4:** Updated fitting when independent variables or their interactions are removed from the full model, separately for each ERP component.

ERP	omit from full model	*r* = *0.5*		*r* = *0.707*		*r* = *1*	
		BF_10_	% pe	BF_10_	% pe	BF_10_	% pe
P1	emo	4.09	±36.08	4.41	±42.66	4.95	±31.39
	size × contr × emo	3.44	±38.49	3.57	±43.81	3.94	±28.79
	size × emo	2.48	±34.55	3.19	±41.86	3.41	±37.58
	contr × emo	2.74	±40.29	3.07	±44.18	3.05	±29.12
	contr	−4.06	±32.33	−3.75	±43.28	−2.50	±43.74
	contr × size	−33.99	±31.33	−33.51	±47.43	−33.17	±31.63
	**size**	**−37.28**	±**33.32**	**−36.82**	±**42.71**	**−36.07**	±**40.72**
N1	contr × emo	3.73	±11.30	4.21	±10.09	4.41	±12.15
	size × emo	3.78	±10.93	4.14	±9.93	4.57	±22.79
	size × contr × emo	3.36	±13.30	3.99	±9.90	3.98	±11.80
	emo	3.35	±10.99	3.81	±9.09	4.16	±13.30
	size	−41.23	±11.44	−40.69	±10.02	−40.55	±12.59
	contr × size	−51.12	±12.86	−50.85	±10.27	−50.72	±12.56
	**contr**	**−127.35**	±**11.26**	**−126.86**	±**10.05**	**−126.73**	±**12.12**
EPN	contr	3.82	±10.41	4.15	±8.03	4.51	±9.08
	contr × size	3.52	±9.78	3.87	±8.63	4.30	±9.25
	size × emo	3.16	±9.70	3.65	±8.40	3.90	±8.23
	contr × emo	2.91	±9.37	3.44	±9.04	3.69	±8.68
	size × contr × emo	0.58	±10.24	0.83	±8.68	1.28	±8.34
	emo	−2.76	±9.97	−2.29	±8.87	−2.09	±9.36
	**size**	**−90.47**	±**10.29**	**−90.19**	±**8.17**	**−89.69**	±**9.36**
LPP	emo	4.28	±32.43	3.87	±37.82	4.56	±37.31
	contr × emo	3.21	±29.68	3.37	±39.06	3.86	±36.84
	size × emo	2.74	±31.16	3.17	±41.51	3.47	±31.21
	size × contr × emo	1.34	±29.15	1.74	±38.07	2.44	±31.29
	contr × size	−3.65	±33.95	−4.03	±40.17	−3.29	±28.96
	contr	−24.35	±30.21	−24.22	±39.80	−23.70	±34.91
	**size**	**−26.55**	±**30.25**	**−26.39**	±**40.68**	**−26.33**	±**30.12**

*Note*. BF_10_ above zero indicates better fitting for the model with omitted factors compared to the full model. Factors are: *emotion* (emo), negative or neutral; *size*, large or small; *contrast* (contr).

To summarize, the amplitude of the P1 seemed to be mostly influenced by font size, contrast, and their interaction – with the lowest values in response to words presented in small font and low contrast –, whereas emotion did not seem to play a role.

### N1

Mean amplitude values of the N1 component were best explained by the *size* × *contrast* × *emotion* interaction model (including all three factors and their interactions) relative to the *null* (BF_10_ > 1.80 × 10^308^). The full model was also 9.96 × 10^16^ times better than the second-best model (additive effects of *size* + *contrast* + *emotion*). Nonetheless, similarly to the P1, follow-up contrasts showed that emotion did not influence N1 amplitude, with evidence favoring the *null* model in all tested comparisons (see Table 3 for details).

Additional top-down model comparisons showed that omitting *contrast* × *emotion* from the full model improved fitting by 67.36 times. Similarly, removing *emotion* × *size*, *size* × *contrast* × *emotion*, and *emotion* also improved fitting by 62.80, 54.05, and 45.15 times, respectively. On the other hand, omitting *size* or *contrast* × *size* lowered the explanatory value of the resulting model by 4.69 × 10^−17^ and 1.21 × 10^22^ times. Finally, omitting the factor *contrast* was maximally detrimental, as it lowered the explanatory value of the resulting model by 1.24 × 10^55^ times.

To summarize, the mean amplitude of the N1 component was reliably modulated by contrast as well as its interaction with size, with lower (i.e., less negative) values following words presented in small font and low contrast. In analogy with the preceding P1 component, emotional valence did not seem to modulate the amplitude of the N1.

### EPN

Mean amplitude of the EPN was best explained by the *size* + *emotion* model, not only relative to the *null* model (BF_10_ > 1.80 × 10^308^) but also compared to the second-best model *size* × *emotion* (32.14). Follow-up paired comparisons investigating emotion-dependent amplitude modulations of this component showed evidence in favor of the *null* model when words were presented in small size and low contrast (BF_10_ = 0.19). However, the *alternative* model had to be preferred over the null when words were presented in small size and high contrast (BF_10_ = 10.07) as well as large size and low contrast (BF_10_ = 5.42). When words were presented in large size and high contrast, evidence remained inconclusive (BF_10_ = 0.53).

Model fitting improved if *contrast*, *contrast* × *size*, *size* × *emotion*, and *contrast* × *emotion* were removed from the full model, whereas removing the *size* × *contrast* × *emotion* interaction only marginally improved fitting. Interestingly, omitting the factor *emotion* decreased the explanatory value of the resulting model by 9.87 times, while removing *size* was much more deleterious (1.48 × 10^39^).

Thus, EPN amplitude was not reliably modulated by contrast but mostly by font size, with larger (i.e., more negative) values in response to words presented in large compared to small font. In addition, emotion had a small but non-negligible additive role, as evidenced by a slight increase in EPN amplitude for unpleasant compared to neutral words when presented in small font and high contrast as well as large font and low contrast.

### LPP

Mean amplitude values of the LPP component were best explained by the *size + contrast + emotion* model (BF_10_ = 4.56 × 10^225^). This model was 450.34 times better than the *full* model. However, paired comparisons showed evidence in favor of the *null* as opposed to the *emotion* model when words were presented in large size and high contrast (BF_10_ = 0.17), small size and high contrast (BF_10_ = 0.18), and large size and low contrast (BF_10_ = 0.31). Evidence was inconclusive when words were presented in small size and low contrast (BF_10_ = 0.79).

Additional analyses showed that removing the factor *emotion* improved fitting by 47.94 times. Similarly, omitting *contrast* × *emotion* (29.08), *size* × *emotion* (23.81), and *size* × *contrast* × *emotion* (5.70) resulted in better fit of the resulting model. Conversely, removing *contrast* × *size* (56.26), *contrast* (3.30 × 10^10^), or *size* (2.89 × 10^11^) was deleterious.

Therefore, in this study, the mean amplitude of the LPP was reliably modulated by additive effects of size and contrast, with overall larger amplitude following words presented in small font and low contrast. Emotional valence did not seem to play a role.

### Exploratory analyses

Visual inspection of the ERP waveforms (left panels of Fig. 1) revealed that the highest peak of the P1 and N1 components changed as a function of experimental condition. This latency shift was not predicted in the pre-registered protocol and, in principle, could be a potential source of bias when analyzing mean amplitude values: for instance, the pre-selected time windows might encompass the whole ERP component in one condition, but only half of it in another one. To overcome this problem, we performed additional exploratory analyses using peak amplitude as dependent variable, with the important caveat that this measure is highly susceptible to noise^53,54^ and the results should therefore be interpreted with caution. We also analyzed peak latency, because this measure could still lead to valuable insights regarding the *speed* at which size and contrast influence event-related electrophysiological signals during emotional word reading. The results of these exploratory analyses – which can be found in the *Supplementary Materials* – did not challenge the main interpretation drawn based on the confirmatory results.

Source estimations were based on significant effects at the scalp level. Source reconstructions of the generators of significant ERP differences were computed and statistically assessed with SPM12^55^. Group inversion^56^ were computed, and the multiple sparse priors algorithm implemented in SPM12 was applied. Inversion results showed strong early visual responses both to size and contrast manipulations. Broad inferior and middle occipital, as well as fusiform responses were found for large words in the P1 and N1 time window. Later, within the EPN time window, additionally significant changes in cortical generators were localized in parietal areas. For high contrast, similarly broad enhanced visual responses were found in the N1 and EPN time window, as well as enhanced motor-related and posterior cingulate cortex activations. Later, in the LPP time window, this effect reversed, and low contrast led to stronger visual activations. Details can be found in the *Supplementary Materials*.

## Discussion

In this study, we orthogonally varied font size, contrast, and emotion content while examining ERP responses associated with sensory and attentional mechanisms. This study was conducted to better understand whether: (i) ERP modulations due to changes in low-level visual features are limited only to font size or can be generalized to other features (here, contrast); (ii) emotional information and low-level features would modulate amplitude *additively* or *interactively*. More generally, we sought to clarify whether sensory gating mechanisms, typically proposed to explain attentional modulations of electrophysiological signals in response to biologically salient pictures, could similarly underlie the enhanced processing of abstract word stimuli carrying a negative emotional meaning. By pre-registering the study and analysis protocol, we minimized biases possibly emerging after observing the study outcome^52,57^.

### Low-level visual features dominate early perceptual processing stages

Font size and contrast were found to explain the observed amplitude changes of the P1 and N1 ERP components, which reflect early stages of stimulus detection and discrimination taking place in the extrastriate visual cortex^3–5^ These results are in line with previous work reporting larger P1 and N1 amplitudes for stimuli with higher contrast and larger size^7–9^. Our experimental design additionally revealed *interactive* effects of contrast and font size on P1 and N1 amplitudes, with lowest amplitudes in response to words presented in small font and low contrast. Moreover, our model comparison approach allowed us to precisely pinpoint the *relative* contribution of low-level features on ERP amplitude modulations. Specifically, for P1 amplitudes, *size* had the largest explanatory value, followed by its interaction with contrast. Conversely, changes in N1 amplitudes were mostly due to *contrast*, followed by its interaction with size. These results point to a possible “hierarchy” among several low-level features during word reading, with size being more salient during initial stimulus detection (P1) while contrast may be more relevant during discrimination processes (N1).

The current results did not reveal early effects of emotional content, in contrast with some studies^49,58,59^, but in accordance with others^29,60,61^. Future work is needed to directly evaluate whether early emotion effects reported in the literature might be contingent upon specific experimental conditions (e.g., lexical vs. semantic vs evaluative tasks). Also, emotion did not interact with either font size or contrast, at variance with similar studies using pictorial stimuli^33,35,36^, indirectly suggesting that biologically relevant pictures may be more salient than words during early stages of stimulus identification and discrimination.

### Independent effects of size and emotion during early attentional selection

Emotional words typically elicit larger EPN compared to neutral words, indicating preferential lexical access due to early attentional selection^26,47,49^. In addition, recent work showed that font size may affect electrophysiological responses to emotional material, as evidenced by more negative EPN amplitude for large pictures and words^36,50^. Our results contribute to this debate in several ways. First, contrast alone does not seem to reliably explain amplitude variations of the EPN during word reading, similar to recent work using emotional and neutral pictures^35^. Second, we partially replicated the findings of Bayer and colleagues^50^ by showing slightly more negative EPN amplitude in response to emotional words when presented in large font, albeit only when contrast was low (right panel of Fig. 1C). These results were obtained using Dutch (instead of German) words, which speaks in favor of the generalizability of these modulatory effects.

We found larger EPN for emotional vs. neutral words also when font size was small and contrast was high. However, in contrast to previous studies using pictures or words^36,50^, no increased EPN amplitude for negative words was observed in response to large, high contrast stimuli. Thus, processing emotional valence while manipulating more than one low-level visual feature gives rise to more complex modulatory effects than previously reported (when only one single low-level feature was changed across conditions). We speculate that, for degraded visual stimuli (e.g., small, low contrast words), there might have been little room for EPN attentional enhancement by negative emotion. Conversely, since large high contrast words were easy to detect, no sensory gain by attentional processes was necessary in this condition. Interestingly, emotional valence seemed to boost brain activity in response to small high contrast as well as large low contrast words, i.e., two conditions in which basic visual information is concurrently *facilitating* and *hindering* recognition. This complex pattern challenges to some degree the idea of an automatic emotion processing at the EPN level, and suggests that enhanced attention to negative emotional words – as captured by the EPN – might depend on the processing efficiency of these low-level features.

### Sustained processing of emotional content may be contingent upon task requests

Previous work has consistently shown larger LPP amplitudes for emotional compared to neutral words^39,40^ likely subtending sustained cognitive processes^28,29^. In our study, font size and contrast modulated LPP amplitude in an additive way, whereas emotion did not seem to play a role.

Several post-hoc explanations can be put forward to account for this result. First, the experimental task may contribute to the systematic modulation of this ERP component. For instance, explicitly requesting participants to pay attention to the semantic content of the words may be more effective in showing emotion-dependent amplitude differences compared to a simple detection task. However, this explanation seems unlikely, since a larger late positivity for emotional as opposed to neutral words has been observed in passive viewing designs^62^, color-naming^63^, lexical decision^48,49,64^, or word identification tasks^65^.

Another source of variation could stem from the task-relevance of the emotional content itself. Some authors argued that emotion captures attention only if (explicitly or implicitly) advantageous for participants to track this feature^66–70^. Indeed, a task that requires evaluating stimulus valence typically elicits stronger emotional modulation of the LPP (e.g., top-down attention to emotion or self-relevance evaluation^19,71^). In addition, Bayer *et al*.^50^ used an *1*-back task to increase compliance. The authors interspersed special trials (identified by a green frame) requiring a button press if the current stimulus was identical to the immediately preceding one. This task requires online maintenance in working memory of the preceding word as well as updating, discrimination, recognition, and comparison with the newly presented word. Thus, constant rehearsal of the previous stimulus is a reasonable and efficient strategy to comply with task demands. In contrast, participants in our study were only required to identify whether the displayed word referred to a color, thereby limiting the processing time needed to complete the task. No updating in working memory was necessary. Therefore, the ERP signal we recorded reflects cognitive processes more consistently related to word reading and not contaminated by working memory components. These arguments notwithstanding, this project was based on Bayer *et al*.^50^ but not meant as its direct replication. Instead, we wished to assess the generalizability of the reported effect using an even simpler experimental paradigm, especially considering that results reported in the literature are not consistent (see *Section 3.1*).

From a different angle, it is also possible that participants’ attention was captured by the high variability in font size and contrast, whose saliency is arguably more powerful than emotional content *per se*. Affective differences might play a negligible role in visual word processing when there is a concurrent, massive variation of these sensory features. When competition occurs between different features, the ones that are the most salient (in this case, size and contrast) would bias attention the most and overshadow any potential effects of weaker ones, here emotion^72^.

It is also possible that we were unable to detect emotion-dependent modulations of electrophysiological activity because being too small, short-lived, or occurring in only partly overlapping time-windows or electrode clusters (or even within non-selected clusters). Recent MEG studies reported emotion-related activity originating from prefrontal generators, not linked to specific components^58,73,74^. However, similar caveats would also apply to earlier studies investigating modulations of early sensory processing at the scalp level.

### Conclusions

The present findings suggest a hierarchical, serial interplay between the processing of low-level visual features and emotional content during word reading. Early perceptual processing was mostly influenced by the interaction between font size and contrast – i.e., smaller P1 and N1 for stimuli harder to discriminate –, whereas emotional content did not seem to be relevant. On the other hand, selective attention allocation was independently affected by font size and emotion: in particular, negative word meaning elicited a larger EPN when stimuli were presented in small font and high contrast. Thus, enhanced attention for negative emotion during word reading at the EPN level is not unconditional, but likely depending on the processing efficiency defined by the combination of low-level features, here with a focus on size and contrast. Later, sustained cognitive processes were sensitive to font size and contrast, presumably more salient than semantic information not only perceptually but also in terms of task-relevance.

## Methods

### Participants

A total of 42 participants were recruited from the student population of Ghent University. They were right-handed, native Dutch-speaking, healthy students, with normal or corrected-to-normal vision. The study protocol was approved by the ethics committee at Ghent University (Faculteit Psychologie en Pedagogische Wetenschappen, Kenmerk 2017/07/Gilles Pourtois), including any relevant details and confirming that the experiment was performed in accordance with relevant guidelines and regulations. Participants were required to sign an informed consent prior to the beginning of the experiment, debriefed at the end of it, and paid € 10 per hour for their participation.

Each dataset was considered eligible for further analyses if the EEG signal – after pre-processing – was judged “clean” based on criteria selected a priori (see *Section 4.4*), as well as demonstrating adequate task engagement based on behavioral performance (see *Section 4.3*). Two datasets were discarded: one due to performance below this threshold, the other because the participant aborted testing. Thus, the final sample consisted of 40 volunteers (all right-handed, median age 23.5, range 19–34, 26 females).

From the 20th participant onward, we monitored Bayes factors (BFs)^75,76^ every 3 participants (because 3 volunteers per day were tested). The *a priori* stopping rules were the following: (i) *statistical rule*: one of the models of interest (see *Section 4.6*) explained amplitude modulations of the components of interest 10 times better than the null model (or vice versa) and 10 times better than the second-best model; (ii) *pragmatic rule*: due to budgetary constraints, we had to stop after a maximum number of 40 participants with acceptable behavioral performance and clean EEG data. A third rule, not explicitly mentioned in the pre-registration protocol but logically following from the pre-registered analysis plan, was that the ERP components of interest had to be reliably different from noise (as confirmed by the procedure highlighted in *Section 4.6*). P1 and N1 were clear even after a few participants, whereas the signal-to-noise ratio of the EPN was generally lower. To ensure a robust identification of this component (as a difference between neutral and negative words, irrespective of size and contrast), we decided to complete data collection using the maximum possible number of participants.

### Stimuli

Emotional and neutral words were selected from a database derived from a large multi-center study^77^. Two-hundred and forty negative and 240 neutral nouns were selected and matched with respect to word length, frequency, power/dominance (i.e., participants judged if words referred to something weak/submissive or strong/dominant), and age of acquisition (see Supplementary Table S1). The whole stimulus set was also rated during pilot testing, to further validate the stimulus selection (see Supplementary Table S2).

### Procedure

Participants were seated in a dimly lit, electrically shielded experimental room, with their head on a chin rest placed approximately 60 cm away from a 19″ CRT screen with resolution of 1,280 × 1,024 pixels. After filling out the informed consent and a short demographic questionnaire, the experiment began. In each trial, a single Dutch word (conveying either unpleasant or neutral content, based on the normative ratings in ref.^77^) was presented on a gray background (RGB values [201, 201, 201]), either in a small or large font (35 vs. 140 pixels; visual angle 3° × 1.1° and 11.8° × 3.6°, respectively) and in high or low contrast (RGB values [0, 0, 0] vs. [191, 191, 191]). This 2 (*emotion*) × 2 (*size*) × 2 (*contrast*) factorial design resulted in the presentation of 480 target words (60 stimuli per condition). For each participant, negative and neutral words were randomly assigned to each of these size and contrast variations. Additionally, 20 words describing colors (e.g., *groen*, i.e., *green* in Dutch) were presented in all size and contrast conditions, resulting in 80 additional probes. To ensure that participants would pay attention to the semantic content of each word, they were required to press the spacebar as soon as they could detect a word referring to a color. Accuracy and response times were recorded to verify task compliance (see *Supplementary Materials*). We decided *a priori* to exclude all participants with accuracy below 80% in any of the four size and contrast conditions, indicating insufficient attention to the words. The total number of 560 words were split in 8 runs of 70 words each. Participants could take a short break in between runs. Each word was presented for 1,000 ms, followed by a variable inter-trial interval between 1,000 and 1,500 ms displaying a fixation cross (70 pixels, RGB values [0, 0, 0]). The whole experiment (including EEG preparation) took approximately 50 minutes. Afterwards, two unrelated exploratory tasks (not part of this manuscript) were administered for approximately 40 minutes. Presentation software v17.2 (www.neurobehavioralsystems.com) was used for stimulus creation and presentation. The commented code is available at https://osf.io/c7g9y/.

### EEG preprocessing

EEG was recorded from 64 Ag/AgCl BioSemi active electrodes (BioSemi, Inc., The Netherlands) at a sampling rate of 256 Hz, online low-pass filtered at 100 Hz. The electrodes were fitted into an elastic cap following the BioSemi position system (i.e., electrode positions are radially equidistant from *Cz*; www.biosemi.com/headcap.htm). Two separate electrodes were used as ground electrodes, a Common Mode Sense active electrode (CMS) and a Driven Right Leg passive electrode (DLR), which form a feedback loop that enables measuring the average potential close to the reference in the A/D-box (www.biosemi.com/faq/cms&drl.htm). Four additional electrodes, placed near the outer canthi of the eyes and above and below the right eye, measured horizontal and vertical eye movements (electro-oculogram, EOG).

Data pre-processing was performed offline with custom scripts in MATLAB (R2015a; The MathWorks, Inc., Natick, MA), using functions included in EEGLAB v14.1.1^78^, ERPLAB v6.1.4^79^, the Signal Processing toolbox (v7.0), and the Statistics and Machine Learning Toolbox (v10.0). The continuous EEG data was assigned electrode coordinates, re-referenced to *Cz* and, after removing linear trends, filtered with separate Hamming windowed sinc FIR filters: (i) *high-pass*: passband edge 0.5 Hz, filter order 1,690, transition bandwidth 0.5 Hz, cutoff frequency (−6 dB) 0.25 Hz; (ii) *low-pass*: passband edge 30 Hz, filter order 114, transition bandwidth 7.4 Hz, cutoff frequency (−6 dB) 33.71 Hz. Flatline or noisy channels, short-time bursts, and ocular movements were detected and corrected via Artifact Subspace Reconstruction^80,81^. Details can be found in the official documentation of the *clean_artifacts* function. For the values assigned to each parameter, see our commented script at https://osf.io/c7g9y. We decided *a priori* to discard any dataset in which the artifact detection procedure identified more than 10 noisy scalp channels. No dataset fulfilled this criterion (the median number of interpolated channels was 4, range 1–10). Noisy channels were interpolated via a spherical spline procedure^82^. Please note that the interpolated channels were mostly identified outside of the clusters selected for the ERP components definition (max interpolated channels in clusters: (2); therefore, any potential distortions of the EEG signal due to interpolation was negligible. Ocular channels were discarded, and the scalp data re-referenced to the average signal. Epochs extending from −200 ms to +1,000 ms time-locked to word onset were created, and baseline correction was applied using the pre-stimulus interval. Finally, 8 grand-averages were computed following each combination of our 2 × 2 × 2 factorial design: (1) negative words, large font, high contrast; (2) negative words, small font, high contrast; (3) negative words, large font, low contrast; (4) negative words, small font, low contrast; (5) neutral words, large font, high contrast; (6) neutral words, small font, high contrast; (7) neutral words, large font, low contrast; (8) neutral words, small font, low contrast.

### Identification of ERP components

The standard approach of selecting electrodes and time windows of the ERP components of interest by visually inspecting the grand-average waveforms can lead to a severe inflation of false positives^53,83^. Furthermore, this approach typically assumes that the ERP components observed in the grand-averaged data are reliably different from noise, but this assumption is seldom verified. To avoid these issues, we computed the grand-average ERP signal across all participants and conditions and conducted repeated measures, two-tailed permutation tests based on the *t*_*max*_ statistic^84^ implemented in the Mass Univariate ERP toolbox v1.25^85,86^:

1. compute the grand average across all trials, conditions, and participants (separately for each electrode and time point). With respect to the EPN, we averaged across all negative and neutral conditions (irrespective of size and contrast) and computed their mean difference (for the rationale, see below);
2. for each time point, compute a *t*-test between this average and a test value of zero (i.e., corresponding to no difference with baseline). The resulting *t*-value is stored and named *t*_*observed*_;
3. randomly permute condition labels (i.e., each observation is either assigned its actual value or zero), calculate the *t*-test, and store its corresponding *t*-value;
4. repeat *step 3* 5,000 times to create a distribution of the possible *t*-values for these data under the null hypothesis;
5. the relative location of *t*_*observed*_ in this empirically generated null distribution provides the *p*-value for the observed data, i.e., how probable the actual difference wave at this specific time point would be if the null hypothesis were true;
6. at each time point, repeat this procedure for each electrode and retain only the highest *t*-value (i.e., *t*_*max*_). The *p*-values for the original observations are derived from the *t*_*max*_ scores.

All timepoints between 0 and 1,000 ms (i.e., 256 timepoints at 256 Hz sampling rate) at all 64 scalp channels were included in the analysis, resulting in 16,384 total comparisons. The resulting differences were considered statistically significant (i.e., desired family-wise error rate kept at ~5%) if they exceeded the *t*_*max*_ of each set of tests.

As already mentioned in the pre-registration protocol, visual inspection of the results of the mass univariate procedure was carried out to minimize Type-II errors (this approach tends to be overly conservative) and ensure that the results would be consistent with well-known characteristics of the ERP components of interest (i.e., polarity, latency, and topography) that have been observed and replicated in the literature. Visual inspection of the localizer data revealed a topography and time window of the EPN that were slightly inconsistent with those reported earlier in the existing literature, i.e., a centroparietal electrode cluster (with positive amplitude values) instead of the typical occipital cluster (with negative amplitude values). Based on this observation, we refined the choice of electrodes and time windows by computing the *t*_*max*_ procedure on negative minus neutral difference waves (see https://osf.io/aev6j/ for the complete procedure in MATLAB). Please note that this approach also minimizes experimenter’s biases, because being performed on data averaged across font size and contrast.

This procedure allowed us to successfully identify the components of interest in the following electrode clusters and time windows post-word onset: (i) *P1*: 66–148 ms, 8 occipito-temporal sensors (P7, P9, PO7, O1, O2, PO8, P8, P10); (ii) *N1*: 150–260 ms, 6 temporal sensors (TP7, P7, P9, TP8, P8, P10); (iii) *EPN*: 300–500 ms, 12 occipito-temporal sensors (P7, P9, PO7, PO3, O1, Oz, Iz, O2, PO4, PO8, P8, P10); (iv) *LPP*: 402–684 ms, 10 parietal sensors (P1, Pz, P2, P4, P6, P8, P10, POz, PO4, PO8). Single-trial amplitude values within the aforementioned time windows and electrode clusters, calculated separately for each participant and condition, were submitted to the statistical analyses described below.

### Statistical analyses of ERP amplitude values

We analyzed the amplitude values of each ERP component (P1, N1, EPN, and LPP) in the framework of model selection using Bayes Factors (BFs)^75,76,87–89^. We used the package *BayesFactor* v0.9.12-2^90^ in *R* v3.4.3 (R Core Team, 2017) to estimate BFs (using Markov-Chain Monte Carlo sampling, 100,000 iterations) for each model of interest versus the null model. The additive models were: (1) *size* + *emotion*; (2) *contrast* + *emotion*; (3) *size* + *contrast* + *emotion*. The interactive models were: (4) *size* × *emotion*; (5) *contrast* × *emotion*; (6) *size* × *contrast* × *emotion*. Participants were included as varying factor, and their variance considered nuisance. Please note that, due to poor model convergence, we could not include stimuli (i.e., words) as random effect, contrary to our pre-registered plan.

To further characterize the direction of the effects, two-tailed Bayesian *t*-tests were calculated to estimate the degree of evidence in favor of a model assuming differences between two specified conditions relative to a model assuming no differences^91,92^. The null hypothesis was specified as a point-null prior (i.e., standardized effect size *δ* = 0), whereas the alternative hypothesis was defined as a Jeffrey-Zellner-Siow (*JZS*) prior, i.e., a folded Cauchy distribution centered around *δ* = 0 with scaling factors of *r* = 1, *r* = 0.707, and *r* = 0.5, to verify the robustness of the results^93^. The most conservative BF was used as reference to decide whether to continue with data collection (see *Section 4.1)*.

### Software used for visualization and statistical analyses

Visualization and statistical analyses were performed using *R*^94^ v3.4.4 via *RStudio*^95^ v1.1.453. We used the following packages (and their respective dependencies):

- data manipulation: *tidyverse*^96^ v1.2.1;
- statistical analyses: *Rmisc*^97^ v1.5, *BayesFactor*^90^ v 0.9.12-4.2;
- visualization: *yarrr*^98^ v0.1.5, *viridis*^99^ v0.5.1, *eegUtils*^100^ v 0.2.0;
- report generation: *pacman*^101^ v0.4.6, *knitr*^102^ v1.20, *here*^103^ v0.1.

## Electronic supplementary material

Supplementary Word File

## Data Availability

Raw and pre-processed data, materials, and analysis scripts are available on https://osf.io/c7g9y/.
