# Supplementary material for: Electrophysiological correlates of the interplay between low-level visual features and emotional content during word reading
Source: Sci Rep. 2018 Aug 15;8:12228. doi: 10.1038/s41598-018-30701-5 (PMC6093870; doi:10.1038/s41598-018-30701-5)
Supplement: Supplementary file 1 — Supplementary Word File [file 41598_2018_30701_MOESM1_ESM.docx]

**Electrophysiological correlates of the interplay between low-level visual features and emotional content**

**during word reading**

**‒**

**Supplementary Materials**

Sebastian Schindler^a,b,c☨*^, Antonio Schettino^a,d☨^, & Gilles Pourtois^a^

^a^ Department of Experimental-Clinical and Health Psychology, Ghent University, Ghent, Belgium

^b^ Department of Psychology, University of Bielefeld, Bielefeld, Germany.

^c^ Institute of Medical Psychology and Systems Neuroscience, University of Muenster, Germany

^d^ Institute for Globally Distributed Open Research and Education (IGDORE)

^☨^ Co-first authors.

^*^ Corresponding author:

Sebastian Schindler

Institute of Medical Psychology and Systems Neuroscience

University of Muenster

48149 Münster (Germany)

Email: sebastian.schindler@ukmuenster.de

# Selection of stimulus material

# Emotional and neutral words were selected from a database derived from a large multicentered study^1^. Two-hundred and forty negative and 240 neutral nouns were selected and matched with respect to word length, frequency, power (feeling weak or dominant in response to a word), and age of acquisition (see *Supplementary Table S1*). The stimulus set was also rated during pilot testing. Nine pilot participants rated half of the negative and neutral nouns (counterbalanced). Participants used self-assessment manikin rating scales^2^ ranging from 1 (highly negative) to 9 (highly positive). For every participant, negative nouns were rated to be more negatively valenced compared to neutral nouns (see *Supplementary Table S2*).

**Supplementary Table S1.** Means and standard deviations (in parenthesis) of normative values of non-emotional features of negative and neutral nouns used in the experiment.

| **Variable** | **Negative nouns (n=240)** | **Neutral nouns (n=240)** | **BF**  **(*r* = .707)** |
| --- | --- | --- | --- |
| Valence | 2.31  (0.35) | 4.02  (0.12) | 2.48x10^97^ |
| Arousal | 4.47  (0.87) | 3.72  (0.56) | 5.84x10^22^ |
| Dominance | 4.21  (0.75) | 4.12  (0.59) | 0.31 |
| Age of Acquisition | 7.99  (1.63) | 7.79  (1.61) | 0.26 |
| Word length | 5.65  (1.42) | 5.59  (1.20) | 0.12 |
| Word frequency  (per million) | 9.73  (13.38) | 9.50  (16.93) | 0.10 |
| Word frequency  (log 10) | 0.61  (0.64) | 0.54  (0.62) | 0.23 |

# 2. Behavioral data

We orthogonally varied font size, contrast, and emotion of pre-selected words while participants were monitoring their semantic content (i.e., they were required to detect rare color-related words in the trial series). The behavioral task was included only to ensure that participants would pay attention to each stimulus presented on screen. Accuracy and response time were analyzed via model comparison using Bayes Factors (see main text for details). The tested models were: (1) main effect of size; (2) main effect of contrast; (3) additive effects of size and contrast; (4) interactive effects of size and contrast. Prior to data collection, we expected no reliable differences in accuracy (because the task was very easy, i.e., ceiling effect) except, perhaps, slightly lower performance for low-contrast small words. With respect to reaction times, we predicted fastest button presses to color words in high contrast and large size, whereas slowest reaction times ought to be observed for low-contrast, small words (i.e., *size x contrast* interaction).

**Supplementary Table S2.** Means and standard deviations (in parenthesis) of valence ratings of negative and neutral nouns by nine pilot participants.

| Participant | Negative nouns | Neutral nouns | *t*_(1, 238)_ | *p*-value | Bayes Factor (*r*=0.707) |
| --- | --- | --- | --- | --- | --- |
| Pilot01(A) | 2.60 (1.18) | 4.78 (1.01) | 15.40 | <.001 | 1.01x10^20^ |
| Pilot02(B) | 2.88 (1.67) | 5.10 (0.65) | 13.61 | <.001 | 1.49x10^29^ |
| Pilot03(A) | 3.42 (0.80) | 4.78 (0.99) | 11.71 | <.001 | 1.01x10^22^ |
| Pilot04(B) | 2.70 (1.58) | 4.91 (0.85) | 13.48 | <.001 | 5.83x10^27^ |
| Pilot05(A) | 2.99 (1.11) | 4.97 (0.50) | 17.65 | <.001 | 8.26x10^22^ |
| Pilot06(B) | 3.49 (0.96) | 5.00 (0.52) | 15.12 | <.001 | 4.35x10^19^ |
| Pilot07(A) | 2.50 (1.37) | 4.82 (0.82) | 15.93 | <.001 | 5.25x10^20^ |
| Pilot08(B) | 2.83 (0.90) | 4.93 (0.55) | 21.83 | <.001 | 5.12x10^27^ |
| Pilot09(A) | 3.74 (1.87) | 4.57 (1.90) | 3.39 | 0.001 | 29.38 |

Note: Scale ranges from 1 (extremely negative) to 9 (extremely positive). Pilots rated half of the used words with counterbalanced sets (A or B).

**2.1 Accuracy**

The proportion of correctly recognized color words was overall very high, as expected given the easy nature of the task (false alarm rate was below one percent, and these trials were discarded from the analyses). Nonetheless, it was lower for small, low-contrast words compared to all other conditions (*Supplementary Table S3*). Bayes factor analysis showed that the *full* interaction model (i.e., including the main effects of size and contrast and their interaction) explained the data *e*^22.53^ = 6.09 x 10^9^ times better than the *null* model and *e*^22.53-13.04^ = *e*^9.49^ = 1.32 x 10^4^ times better than the second-best model (i.e., including only main effects) (*Supplementary Table S4*. For ease of readability, only results obtained with JZS priors with location *δ* = 0 and scaling factor *r* = 0.707 are reported in the main text. Results obtained with other scaling factors can be found in the respective tables).

**Supplementary Table S3.** Means and standard deviations (in parenthesis) of accuracy and reaction times in the color word detection task.

| **Dependent Variable** | **Large**  **High contrast** | **Large**  **Low contrast** | **Small**  **High contrast** | **Small**  **Low contrast** |
| --- | --- | --- | --- | --- |
| Accuracy  (proportion correct) | .97  (0.04) | .97  (0.04) | .97  (0.03) | .90  (0.06) |
| Reaction Times  (in milliseconds) | 573.26  (25.29) | 597.42  (24.61) | 583.52  (19.70) | 683.21  (37.45) |

Paired comparisons showed lower accuracy for words presented in low compared to high contrast when font size was small (BF_10_ = *e^14.54^* = 2.06 x 10^6^). Low contrast words were also recognized less accurately when presented in small vs. large font (BF_10_ = 5.31 x 10^4^). Conversely, the null model explained the data better than the models hypothesizing differences between words in large font presented in low vs. high contrast (BF_10_ = 0.19) or low contrast presented in large vs. small font (BF_10_ = 0.17) (see *Supplementary Table S5*).

**Supplementary Table S4.** Model comparisons for accuracy and reaction times.

| **Dependent variable** | **model** | ***r = 0.5*** | | ***r = .707*** | | ***r = 1*** | |
| --- | --- | --- | --- | --- | --- | --- | --- |
|  |  | **BF_10_** | **% pe** | **BF_10_** | **% pe** | **BF_10_** | **% pe** |
| **Accuracy** | **size x contr** | **22.48** | **±0.92** | **22.53** | **±0.91** | **22.24** | **±0.98** |
|  | size + contr | 13.12 | ±0.98 | 13.02 | ±1.00 | 12.73 | ±1.07 |
|  | contr | 5.29 | ±1.07 | 5.26 | ±1.08 | 5.10 | ±1.11 |
|  | size | 3.24 | ±1.11 | 3.16 | ±1.14 | 2.97 | ±1.18 |
| **Reaction Times** | **size x contr** | **83.12** | **±1.52** | **83.80** | **±1.10** | **84.23** | **±0.90** |
|  | size + contr | 57.86 | ±0.88 | 58.20 | ±0.74 | 58.37 | ±0.69 |
|  | contr | 32.21 | ±0.63 | 32.34 | ±0.59 | 32.39 | ±0.57 |
|  | size | 19.01 | ±0.59 | 19.04 | ±0.58 | 18.96 | ±0.60 |

*Note.* Bayes factors (BF_10_, on *log* scale) and percentage of proportional errors (% pe) for each model relative to the null, obtained by using JZS priors with different scaling factors (see *Section 4.8* in the main text for details). The model best explaining the data for each dependent variable is highlighted in bold.

**2.2 Reaction times**

Reaction times for correct responses were substantially slower for small, low-contrast words compared to all other conditions (*Supplementary Table S3*). Bayesian analysis showed that the full *size x contrast* interaction model explained the observed data 2.48 x 10^36^ times better than the *null* model and 1.31 x 10^11^ times better than the *size + contrast* model (*Supplementary Table S4*).

Paired comparisons (see *Supplementary Table S5*) showed slower RTs for small words presented in low compared to high contrast (BF_10_ = 5.55 x 10^14^). The same pattern was observed when stimuli were presented in large font size (BF_10_ = 1.35 x 10^3^). The post-hoc comparison within low contrast showed that small compared to large words were recognized more slowly (BF_10_ = 1.19 x 10^11^). Finally, the difference between high-contrast words in large vs. small font was not conclusive (BF_10_ = 1.70).

**Supplementary Table S5.** Post-hoc comparisons for reaction times and accuracy.

| **Dependent variable** | **post-hoc comparison** | ***r = 0.5*** | | ***r = .707*** | | ***r = 1*** | |
| --- | --- | --- | --- | --- | --- | --- | --- |
|  |  | **BF_10_** | **% pe** | **BF_10_** | **% pe** | **BF_10_** | **% pe** |
| **Accuracy** | **small size, high vs. low contrast** | **14.35** | **±0.00** | **14.54** | **±0.00** | **14.63** | **±0.00** |
|  | **low contrast, large vs small size** | **10.75** | **±0.00** | **10.88** | **±0.00** | **10.91** | **±0.00** |
|  | large size, high vs. low contrast | -1.36 | ±0.00 | -1.66 | ±0.00 | -1.98 | ±0.00 |
|  | high contrast, large vs. small size | -1.46 | ±0.00 | -1.77 | ±0.00 | -2.09 | ±0.00 |
| **Reaction Times** | **small size, high vs. low contrast** | **33.65** | **±0.00** | **33.95** | **±0.00** | **34.22** | **±0.00** |
|  | **low contrast, large vs small size** | **25.23** | **±0.00** | **25.50** | **±0.00** | **25.71** | **±0.00** |
|  | **large size, high vs. low contrast** | **7.15** | **±0.00** | **7.21** | **±0.00** | **7.16** | **±0.00** |
|  | high contrast, large vs. small size | 0.71 | ±0.00 | 0.53 | ±0.00 | 0.29 | ±0.00 |

*Note.* Bayes factors (BF_10_, on *log* scale) and percentage of proportional errors (% pe) for each model assuming pairwise differences between conditions relative to the null model (details are provided in *Section 4.8*). BF_10_ above zero indicates better fitting for the alternative compared to the null model. Post-hoc comparisons in favor of a difference are highlighted in bold.

# 3. Peak amplitude and latency analysis of P1 and N1 components

As mentioned in *Section 2.3* of the main text, visual inspection of the waveforms revealed that the peak of the P1 and N1 components were shifted as a function of experimental condition (see also left panels of *Figure 2*). Therefore, we explored peak amplitude and latency of these components to ensure that the results of the confirmatory analyses would not be contaminated by this potential confound.

Peaks were scored as the positive value (negative for N1) larger than the 3 timepoints on either side of this value, corresponding to ~10 ms at 256 Hz sampling rate. Peak values reflected the amplitude at these points, whereas peak latency was the time (in milliseconds) in which the peaks occurred (see *Supplementary Figure 1* and *Supplementary Table S6*).

**Supplementary Table S6:** Means and standard deviations (in parenthesis) of peak amplitude (in µV) and peak latency (in ms) values of the P1 and N1 components.

| measure | component | large | | | | small | | | |  |
| --- | --- | --- | --- | --- | --- | --- | --- | --- | --- | --- |
|  |  | high | | low | | high | | low | |  |
|  |  | negative | neutral | negative | neutral | negative | neutral | negative | neutral |  |
| peak | P1 | 3.29 (1.06) | 3.35 (1.03) | 3.14 (0.87) | 3.00 (0.94) | 2.91 (0.83) | 2.67 (0.94) | 1.47 (1.22) | 1.30 (1.66) |  |
| amplitude | N1 | -3.51 (1.05) | -3.50 (1.13) | -3.07 (0.71) | -3.05 (0.84) | -3.24 (0.88) | -3.30 (1.09) | -0.99 (1.72) | -1.24 (1.31) |  |
| peak | P1 | 102 (15.29) | 104 (13.59) | 126 (17.39) | 124 (21.13) | 117 (13.40) | 113 (9.93) | 117 (22.47) | 115 (27.01) |  |
| latency | N1 | 190 (23.40) | 188 (19.40) | 211 (17.40) | 202 (20.10) | 196 (17.50) | 199 (21.20) | 214 (32.10) | 215 (30.50) |  |

## **3.1 P1**

Based on the results of the mass univariate analysis (see *Section 4.5* in the main text), we bounded our search to the time window between 66 and 148 ms after stimulus onset at electrodes *P7*, *P9*, *PO7*, *O1*, *O2*, *PO8*, *P8*, and *P10*.

### ***3.1.1 Peak amplitude***

Peak amplitude values of the P1 component were best explained by the *size x contrast x emotion* model, with a BF of 3.31 x 10^48^ relative to the *null* model. This model was also 5.37 times better than the second best model assuming additive effects of size, contrast, and emotion (see *Supplementary Table S7*). However, follow-up contrasts showed no reliable amplitude differences as a function of emotional content (see *Supplementary Table S8*).

Additional top-down model comparison (see *Supplementary Table S9*) showed that omitting *contrast x emotion*, *emotion*, *size x emotion*, and *size x contrast x emotion* would improve fitting by 7.77, 7.10, 6.62, and 5.16 times, respectively. Conversely, omitting *contrast x size* or *contrast* would lower the explanatory value of the resulting model by 1.51 x 10^3^ and 1.75 x 10^7^ times, respectively. Finally, removing the factor *size* was maximally detrimental, as it would lower the explanatory value of the resulting model by 1.31 x 10^13^ times.

These results are in line with the confirmatory results using mean amplitude values as dependent variable: emotion penalized the models in which it was included, whereas font size, contrast, and their interaction seemed to be mostly responsible for the amplitude variations of the P1 (i.e., less positive following low contrast, small sized words).

**
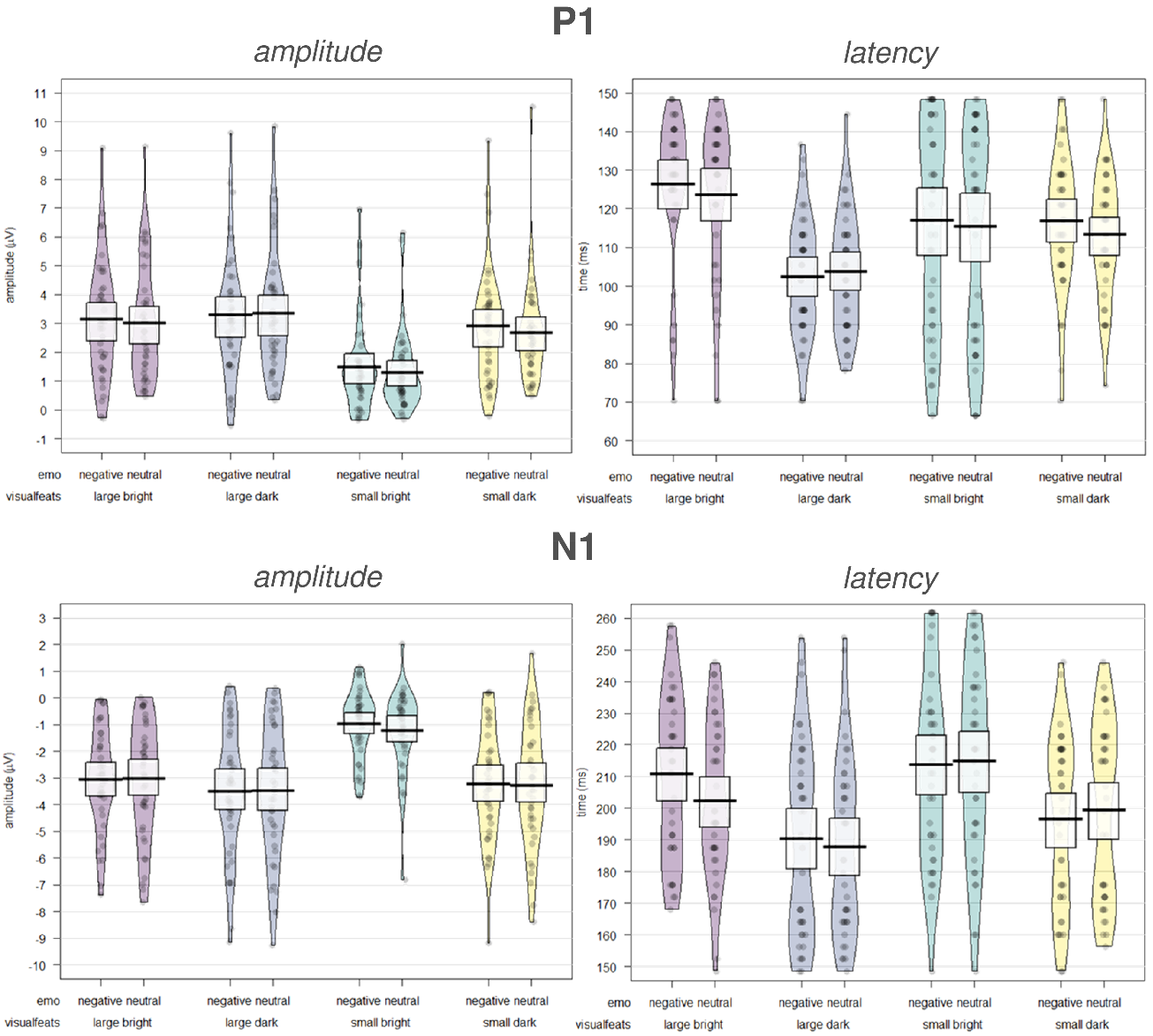
**

**Supplementary Figure 1. Peak amplitude and latency values of the P1 and N1 components.** Amplitude values for each participant (gray dots) and experimental condition. Mean amplitude values are marked by horizontal black lines and 95% Bayesian highest density interval (HDI) are displayed as white boxes.

### ***3.1.2 Peak latency***

Peak latencies were best explained by the *size x contrast x emotion* model, with a BF_10_ of 5.77 x 10^3^ relative to the *null* model. The winning model was only 3.74 times better than the second-best model, i.e., *contrast + emotion* (*Supplementary Table S7*). Follow-up contrasts showed no emotion-dependent latency differences (*Supplementary Table S8*).

Additional model comparison showed improved fitting when omitting *emotion*, *size*, *contrast x emotion*, *size x emotion*, and *size x contrast x emotion* (*Supplementary Table S9*). On the other hand, omitting *contrast x size* or *contrast* would lower the explanatory value of the resulting model by 1.20 x 10^4^ and 8.63 x 10^6^ times, respectively.

These results show that emotion did not affect P1 latency, whereas contrast seemed the strongest predictor, especially when interacting with size. P1 peaked earlier in response to words displayed in large font size and high contrast compared to all other conditions.

**Supplementary Table S7:** Model comparisons, separately for peak amplitude and latency, for the P1 and N1 ERP components.

| measure | component | model | *r = 0.5* | | *r = .707* | | *r = 1* | |
| --- | --- | --- | --- | --- | --- | --- | --- | --- |
|  |  |  | BF_10_ | % pe | BF_10_ | % pe | BF_10_ | % pe |
| peak | P1 | size x contr x emo | 113.07 | ±2.10 | 111.72 | ±2.21 | 109.99 | ±2.50 |
|  |  | size + contr + emo | 110.40 | ±0.97 | 110.04 | ±1.00 | 109.45 | ±1.07 |
|  |  | size + emo | 94.89 | ±0.87 | 94.57 | ±0.87 | 94.13 | ±0.89 |
|  |  | size x emo | 93.28 | ±1.09 | 92.66 | ±1.10 | 91.86 | ±1.16 |
|  |  | contr + emo | 82.29 | ±0.86 | 81.85 | ±0.89 | 81.29 | ±0.94 |
|  |  | contr x emo | 80.53 | ±1.06 | 79.76 | ±1.12 | 78.91 | ±1.18 |
| amplitude | N1 | size x contr x emo | 132.47 | ±2.24 | 131.36 | ±2.20 | 129.88 | ±2.34 |
|  |  | size + contr + emo | 120.50 | ±1.02 | 120.20 | ±1.00 | 119.72 | ±1.04 |
|  |  | contr + emo | 96.74 | ±0.88 | 96.44 | ±0.87 | 96.02 | ±0.91 |
|  |  | contr x emo | 95.03 | ±1.09 | 94.37 | ±1.09 | 93.63 | ±1.14 |
|  |  | size + emo | 86.88 | ±0.85 | 86.46 | ±0.88 | 85.97 | ±0.91 |
|  |  | size x emo | 85.22 | ±1.06 | 84.50 | ±1.11 | 83.70 | ±1.18 |
| peak | P1 | size x contr x emo | 10.49 | ±4.44 | 8.66 | ±4.74 | 6.63 | ±4.13 |
|  |  | contr + emo | 7.82 | ±1.88 | 7.37 | ±1.98 | 6.86 | ±2.08 |
|  |  | contr x emo | 6.12 | ±2.31 | 5.33 | ±2.39 | 4.45 | ±2.68 |
|  |  | size + contr + emo | 5.96 | ±2.28 | 5.28 | ±2.44 | 4.38 | ±2.51 |
|  |  | size + emo | -4.88 | ±2.47 | -5.49 | ±2.49 | -6.16 | ±2.53 |
|  |  | size x emo | -6.56 | ±2.88 | -7.52 | ±2.98 | -8.52 | ±3.22 |
| latency | N1 | size + contr + emo | 26.64 | ±1.16 | 25.99 | ±1.24 | 25.18 | ±1.32 |
|  |  | contr + emo | 24.24 | ±1.01 | 23.84 | ±1.05 | 23.31 | ±1.11 |
|  |  | cont x emo | 22.71 | ±1.25 | 21.96 | ±1.32 | 21.13 | ±1.37 |
|  |  | size x contr x emo | 21.12 | ±2.64 | 19.27 | ±2.95 | 17.09 | ±3.03 |
|  |  | size + emo | 11.10 | ±1.15 | 10.52 | ±1.20 | 9.91 | ±1.22 |
|  |  | size x emo | 10.11 | ±1.38 | 9.24 | ±1.45 | 8.28 | ±1.55 |

## **3.2 N1**

Following the results of the mass univariate analysis, we limited our search to the time window between 150 and 260 ms post-stimulus onset at electrodes T*P7*, *P7*, *P9*, *TP8*, *P8*, and *P10*.

### ***3.2.1 Peak amplitude***

Peak amplitude values were best explained by the *size x contrast x emotion* model, with a BF_01_ of 1.12 x 10^57^ relative to the *null* model. This model was also 7.03x 10^4^ times better than the second-best model assuming independent effects of *size*, *contrast*, and *emotion* (*Supplementary Table S7*). However, follow-up contrasts showed no emotion-dependent amplitude differences (*Supplementary Table S8*).

Additional model comparison showed that omitting *emotion*, *contrast x emotion*, *size x emotion*, and *size x contrast x emotion* improved fitting by 10.10, 7.77, 6.75, and 5.64 times, respectively. On the other hand, omitting *contrast x size*, *size*, or *contrast* would lower the explanatory value of the resulting model by 2.04 x 10^7^, 6.76 x 10^11^, and 4.71 x 10^16^ times, respectively (*Supplementary Table S9*).

Analogous to the results on mean amplitude reported in the main text, the peak amplitude of the N1 component was modulated by contrast as well as its interaction with size, but not by emotional valence. N1 peaks were less negative following words in low contrast and small font size compared to all other conditions.

**Supplementary Table S8:** Post-hoc comparisons, separately for each ERP component.

| measure | component | post hoc comparison | *r = 0.5* | | *r = .707* | | *r = 1* | |
| --- | --- | --- | --- | --- | --- | --- | --- | --- |
|  |  |  | BF_10_ | % pe | BF_10_ | % pe | BF_10_ | % pe |
| peak | P1 | large size, high contrast, negative vs. neutral | -1.33 | ±0.00 | -1.63 | ±0.00 | -1.95 | ±0.00 |
|  |  | small size, high contrast, negative vs. neutral | -0.08 | ±0.00 | -0.30 | ±0.00 | -0.57 | ±0.00 |
|  |  | large size, low contrast, negative vs. neutral | -1.22 | ±0.00 | -1.52 | ±0.00 | -1.83 | ±0.00 |
|  |  | small size, low contrast, negative vs. neutral | -1.19 | ±0.00 | -1.49 | ±0.00 | -1.80 | ±0.00 |
| amplitude | N1 | large size, high contrast, negative vs. neutral | -1.46 | ±0.00 | -1.77 | ±0.00 | -2.09 | ±0.00 |
|  |  | small size, high contrast, negative vs. neutral | -1.40 | ±0.00 | -1.70 | ±0.00 | -2.02 | ±0.00 |
|  |  | large size, low contrast, negative vs. neutral | -1.46 | ±0.00 | -1.76 | ±0.00 | -2.09 | ±0.00 |
|  |  | small size, low contrast, negative vs. neutral | -1.05 | ±0.00 | -1.33 | ±0.00 | -1.64 | ±0.00 |
| peak | P1 | large size, high contrast, negative vs. neutral | -1.32 | ±0.00 | -1.62 | ±0.00 | -1.94 | ±0.00 |
|  |  | small size, high contrast, negative vs. neutral | -0.44 | ±0.00 | -0.68 | ±0.00 | -0.96 | ±0.00 |
|  |  | large size, low contrast, negative vs. neutral | -1.29 | ±0.00 | -1.59 | ±0.00 | -1.90 | ±0.00 |
|  |  | small size, low contrast, negative vs. neutral | -1.43 | ±0.00 | -1.74 | ±0.00 | -2.06 | ±0.00 |
| latency | N1 | large size, high contrast, negative vs. neutral | -1.29 | ±0.00 | -1.59 | ±0.00 | -1.91 | ±0.00 |
|  |  | small size, high contrast, negative vs. neutral | -1.21 | ±0.00 | -1.50 | ±0.00 | -1.82 | ±0.00 |
|  |  | large size, low contrast, negative vs. neutral | 0.50 | ±0.00 | 0.31 | ±0.00 | 0.07 | ±0.00 |
|  |  | small size, low contrast, negative vs. neutral | -1.45 | ±0.00 | -1.75 | ±0.00 | -2.08 | ±0.00 |

***3.2.2 Peak latency***

Peak latencies of the N1 were best explained by the model with additive effects of *size*, *contrast*, and *emotion* (*Supplementary Table S7*), which was also 8.58 times better than the second-best model (*contrast* + *emotion*). Follow-up contrasts (*Supplementary Table S8*) showed evidence in favor of the *null* model for words presented in small size and low contrast (BF_10_ = 0.17), large size and high contrast (BF_10_ = 0.20), and small size and high contrast (BF_10_ = 0.22), whereas differences in large size and low contrast were inconclusive (BF_10_ = 1.36).

Additional model comparison showed improved fitting when omitting *emotion*, *contrast x size*, *contrast x emotion*, *size x contrast x emotion*, and *size x emotion* (*Supplementary Table S9*). Conversely, omitting *size* would lower the explanatory value of the resulting model by 8.94 times. Omitting *contrast* would be maximally detrimental, as it would lower the explanatory value of the resulting model by 6.14 x 10^6^ times.

These results show that emotion did not affect N1 latency, whereas size and contrast modulated it independently, with delayed peaks following low contrast and small sized words.

**Supplementary Table S9:** Updated fitting when factors are removed from the full model, separately for each ERP component.

| measure | component | omit from full model | *r = 0.5* | | *r = .707* | | *r = 1* | |
| --- | --- | --- | --- | --- | --- | --- | --- | --- |
|  |  |  | BF_10_ | % pe | BF_10_ | % pe | BF_10_ | % pe |
| peak | P1 | contr x emo | 1.74 | ±2.56 | 2.05 | ±2.89 | 2.39 | ±3.39 |
|  |  | emo | 1.68 | ±2.53 | 1.96 | ±2.86 | 2.31 | ±3.34 |
|  |  | size x emo | 1.58 | ±2.58 | 1.89 | ±2.87 | 2.20 | ±3.28 |
|  |  | size x contr x emo | 1.36 | ±2.57 | 1.64 | ±2.87 | 1.99 | ±3.32 |
|  |  | contr x size | -7.32 | ±2.69 | -7.32 | ±2.96 | -7.19 | ±3.42 |
|  |  | contr | -16.71 | ±2.67 | -16.68 | ±3.09 | -16.56 | ±3.41 |
|  |  | size | -30.01 | ±2.70 | -30.20 | ±2.93 | -30.16 | ±3.37 |
| amplitude | N1 | emo | 1.94 | ±2.83 | 2.31 | ±2.69 | 2.65 | ±2.94 |
|  |  | contr x emo | 1.69 | ±2.83 | 2.05 | ±2.70 | 2.39 | ±2.94 |
|  |  | size x emo | 1.58 | ±2.83 | 1.91 | ±2.66 | 2.29 | ±2.95 |
|  |  | size x contr x emo | 1.37 | ±2.86 | 1.73 | ±2.70 | 2.04 | ±2.98 |
|  |  | contr x size | -16.78 | ±2.89 | -16.83 | ±2.80 | -16.80 | ±3.00 |
|  |  | size | -27.19 | ±2.98 | -27.24 | ±2.84 | -27.28 | ±3.01 |
|  |  | contr | -38.21 | ±2.88 | -38.39 | ±2.75 | -38.45 | ±3.11 |
| peak | P1 | emo | 1.80 | ±5.20 | 2.21 | ±5.41 | 2.46 | ±6.60 |
|  |  | size | 1.84 | ±5.40 | 2.17 | ±5.14 | 2.37 | ±6.52 |
|  |  | contr x emo | 1.75 | ±5.46 | 2.06 | ±5.32 | 2.28 | ±6.55 |
|  |  | size x emo | 1.69 | ±5.33 | 1.98 | ±5.22 | 2.23 | ±6.55 |
|  |  | size x contr x emo | 1.17 | ±5.35 | 1.54 | ±5.16 | 1.83 | ±6.56 |
|  |  | contr x size | -9.11 | ±6.10 | -9.03 | ±6.28 | -9.01 | ±7.21 |
|  |  | contr | -11.81 | ±5.64 | -11.66 | ±6.02 | -11.60 | ±6.93 |
| latency | N1 | emo | 1.93 | ±3.22 | 2.23 | ±3.69 | 2.52 | ±3.97 |
|  |  | contr x size | 1.79 | ±3.22 | 2.12 | ±3.89 | 2.36 | ±3.92 |
|  |  | contr x emo | 1.58 | ±3.25 | 1.86 | ±3.70 | 2.15 | ±3.97 |
|  |  | size x contr x emo | 1.36 | ±3.19 | 1.69 | ±3.69 | 1.98 | ±3.97 |
|  |  | size x emo | 0.88 | ±3.24 | 1.18 | ±3.77 | 1.44 | ±3.98 |
|  |  | size | -2.39 | ±3.62 | -2.19 | ±3.74 | -1.91 | ±4.53 |
|  |  | contr | -15.65 | ±3.70 | -15.63 | ±4.04 | -15.51 | ±4.38 |

# 4. Estimation of the neural sources

Source reconstruction of the generators of significant ERP differences were computed and statistically assessed with SPM12 for EEG^3^. First, a realistic boundary element head model (BEM) was derived from SPM’s template head model based on the Montreal Neurological Institute (MNI) brain. Electrode positions were then transformed to match the template head, which is thought to generate reasonable results even when an individual’s head differs from the template. Average electrode positions as provided by BioSemi were co-registered with the cortical mesh template for source reconstruction. This cortical mesh was used to calculate the forward solution. The inverse solution was calculated from 0 to 1,000 ms after word onset. Group inversion^4^ were computed, and the multiple sparse priors algorithm implemented in SPM12 was applied. This method allows activated sources to vary in the degree of activity but restricts the activated sources to be the same in all participants^44^. This has been found to result in more robust source estimations compared to single-subject matrix inversion^4^.

Statistical analyses in source space were performed for significant scalp effects by applying the same time window as in scalp space (see main text). 3D reconstructions were generated as NIFTI images (voxel size = 2mm*2mm*2mm) and smoothed using an 8mm full-width half-maximum filter. Statistical comparisons reported for source space were restricted to time windows that revealed significant differences on the scalp. Similar to previous studies^5^, we described statistical differences in source activity of voxels differing at least at an uncorrected threshold of *p* < .005 and a minimum of 15 significant voxels per cluster. In addition, results using family-wise error (FWE) corrected threshold of *p* < .05 and a minimum of 15 significant voxels per cluster are reported in all tables. The identification of activated brain regions was performed using the LONI atlas^6^.

## ***4.1 P1***

Follow-up source estimations showed stronger activations for large words in visual areas including the inferior and middle occipital gyrus as well as in the fusiform gyrus (see *Supplementary Figure 2* and *Supplementary Table S10*). Activations were stronger in the left hemisphere, including a small activation in the left middle frontal gyrus.


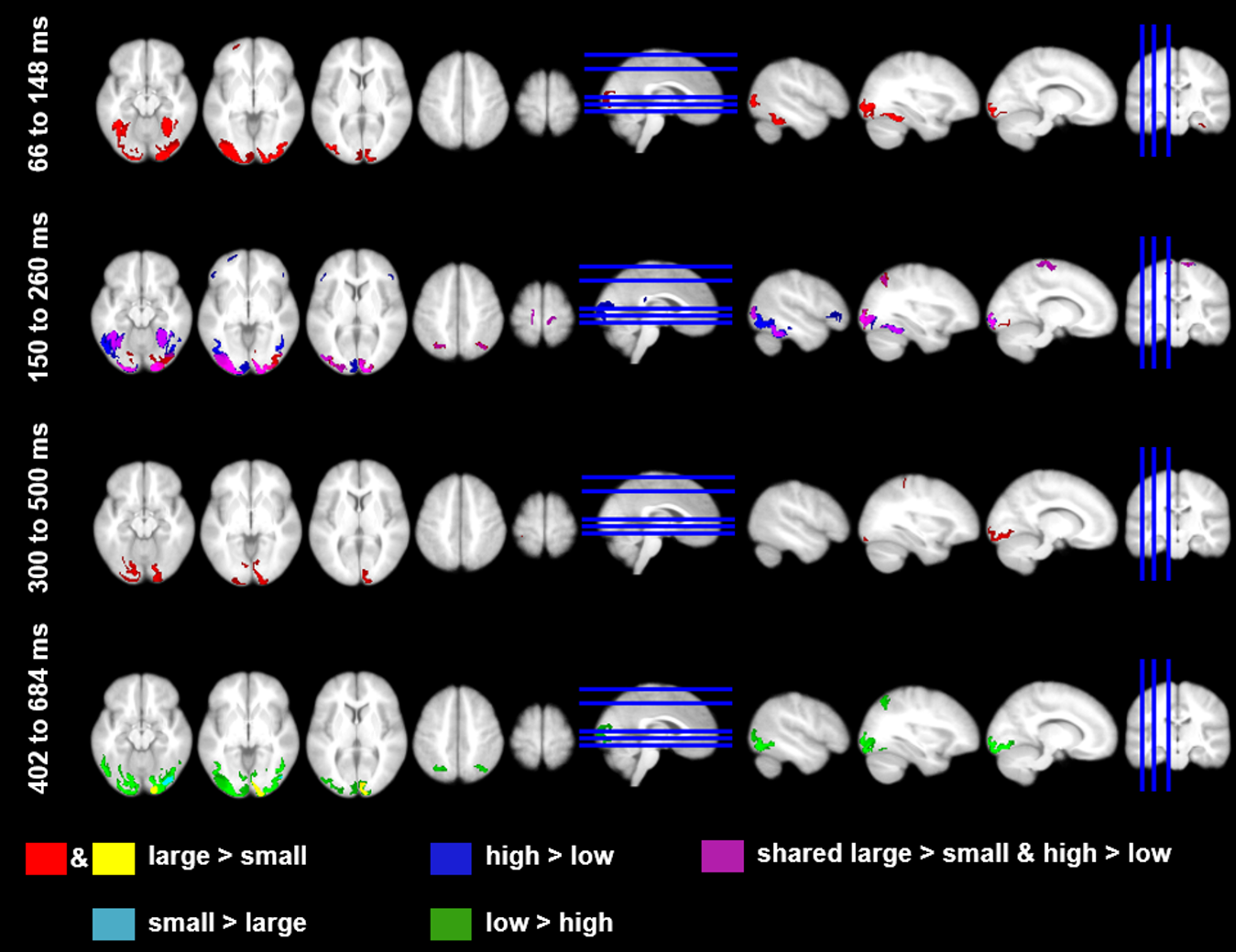
**Supplementary Figure 2.** Source estimations for size and contrast effects.

## ***4.2 N1***

Explorations in source space for contrast and size effects showed that the neural generators were found to be stronger for large size words in inferior and middle occipital gyri, angular and fusiform gyri, as well as the left temporal gyrus and precentral areas. High contrast elicited even broader activations in inferior and middle occipital areas, angular and fusiform gyri, precentral and left temporal regions. The pattern was found to be more left-lateralized, including additional activations in left inferior frontal and middle activations were observed.

## ***4.3 EPN***

Neural generators were found to be stronger for large size words in occipital and parietal regions, including the bilateral superior parietal gyri, the right cuneus and left lingual gyrus.

## ***4.4 LPP***

Explorations in source space showed that large sizes led to enhanced activations in right lingual gyrus while small sizes engaged the inferior occipital gyrus. In contrast to previous time windows, low-contrast words activated broad visual activations, including bilateral inferior and middle occipital as well as angular gyri.

**Supplementary Table S10:** Explorations in source space for size and contrast main effects.

| **cluster-level** | **peak-level** | | | **MNI coordinates** | | | **LONI** |
| --- | --- | --- | --- | --- | --- | --- | --- |
| Number of significant voxels | peak  *t* (1, 316) | | peak  *p*-unc | x (mm) | y (mm) | z (mm) | area |
| *P1 time window (66-148 ms)* | | | | | | | |
| *Size: large>small* | | | | | | | |
| 2,216 (1,016^a^) | 7.08 | <.001 | | -24 | -92 | -6 | L middle occipital gyrus |
| 1,336 (667^a^) | 5.87 | <.001 | | 24 | -96 | -16 | L inferior occipital gyrus |
| 714 (449^a^) | 5.40 | <.001 | | 42 | -38 | -24 | R fusiform gyrus |
| 32 | 3.22 | =.001 | | -26 | 58 | -4 | L middle frontal gyrus |
| 18 | 3.12 | =.001 | | 32 | -18 | -30 | R fusiform gyrus |
| *N1 time window (150-260 ms)* | | | | | | | |
| *Size: large>small* | | | | | | | |
| 1,165 (374^a^) | 7.56 | <.001 | | -26 | -98 | -4 | L middle occipital gyrus |
| 1,532 (594^a^) | 7.08 | <.001 | | 22 | -100 | -6 | R middle occipital gyrus |
| 699 | 4.24 | <.001 | | -48 | -50 | -26 | L inferior temporal gyrus |
| 627 | 4.18 | <.001 | | 42 | -38 | -24 | R fusiform gyrus |
| 144 | 3.72 | <.001 | | -10 | -24 | 74 | L precentral gyrus |
| 150 | 3.71 | <.001 | | 12 | -24 | 70 | R precentral gyrus |
| 92 | 3.63 | <.001 | | -12 | -80 | -12 | L inferior occipital gyrus |
| 233 | 3.61 | <.001 | | 38 | -66 | 48 | R angular gyrus |
| 235 | 3.57 | <.001 | | -32 | -68 | 50 | L angular gyrus |
| *Contrast: high>low* | | | | | | | |
| 4,175 (1,782^a^) | 8.72 | <.001 | | -50 | -50 | -24 | L inferior temporal gyrus |
| 1,300 (628^a^) | 6.08 | <.001 | | 40 | -64 | -18 | R inferior occipital gyrus |
| 151 | 3.67 | <.001 | | 12 | -24 | 70 | R precentral gyrus |
| 142 | 3.64 | <.001 | | -12 | -24 | 74 | L precentral gyrus |
| 113 | 3.17 | =.001 | | -50 | 22 | -2 | L inferior frontal gyrus |
| 49 | 3.08 | =.001 | | -26 | 58 | -4 | L middle frontal gyrus |
| 76 | 3.08 | =.001 | | 52 | 26 | 0 | R inferior frontal gyrus |
| 144 | 2.96 | =.002 | | 34 | -66 | 46 | R angular gyrus |
| 139 | 2.94 | =.002 | | -32 | -68 | 50 | L angular gyrus |
| 73 | 2.94 | =.002 | | 0 | -36 | 18 | cingulate cortex |
| *EPN time window (300-500 ms)* | | | | | | | |
| *Size: large>small* | | | | | | | |
| 848 (146^a^) | 5.68 | <.001 | | 10 | -82 | -8 | R cuneus |
| 706 | 4.53 | <.001 | | -12 | -82 | -12 | L lingual gyrus |
| 69 | 3.00 | =.001 | | 40 | -36 | 60 | R superior parietal gyrus |
| 68 | 2.98 | =.001 | | -40 | -38 | 60 | L superior parietal gyrus |
| *LPP time window (402-684 ms)* | | | | | | | |
| *Size: large>small* | | | | | | | |
| 452 | 3.83 | <.001 | | 12 | -92 | -8 | R lingual gyrus |
| *Size: small>large* | | | | | | | |
| 132 | 2.81 | =.002 | | 40 | -82 | -16 | R inferior occipital gyrus |
| *Contrast: low>high* | | | | | | | |
| 4,731 (1,902^a^) | 8.50 | <.001 | | 24 | -90 | -14 | R inferior occipital gyrus |
| 298 | 4.05 | <.001 | | 34 | -66 | 46 | R angular gyrus |
| 294 | 4.03 | <.001 | | -32 | -68 | 50 | L angular gyrus |

Notes. ^a^Resulting cluster size when FWE-corrected threshold of *p* < .05 (≥15 significant voxels) was used. No. of sig. voxel = number of voxel which differ significantly between both conditions. Peak *p*-unc = uncorrected *p*-value. For each significant peak, respective coordinates (x, y and z) are displayed in MNI space. A cluster may exhibit more than one peak, while only the largest peak is reported. Area = peak-level brain region as identified by the LONI atlas. R / L = laterality right or left.

# 5. Discussion

The exploratory analysis on peak amplitude values of the P1 and N1 components do not challenge the results of the confirmatory analyses using mean amplitude (see main text). Both the P1 and N1 peaks were most sensitive to changes in font size and contrast, whereas differences in the emotional content of the words did not seem to modulate these ERP components. Similarly, latency analysis revealed delayed P1 and N1 peaks for perceptually challenging stimuli ‒ i.e., with low contrast and small font size ‒, but no influence of emotional content. The combined results of our confirmatory and exploratory analyses converge in indicating no reliable emotion-dependent modulation of early ERP components, in contrast with some studies^7–9^, but in accordance with others^10–12^.

Source estimations were based on significant effects in scalp space. Accordingly, these inversion results show strong and early visual responses both to size and contrast manipulations. Responses in broad inferior and middle occipital as well as fusiform areas were found for large words in the P1 and N1 time window. Later, within the EPN time window, additionally significant changes in cortical generators were located to parietal areas. For high contrast, similarly broad enhanced visual responses were found in the N1 and EPN time window, but also enhanced motor-related and cingulate cortex activations. Later, in the LPP time window, this effect reversed, whereby low contrast led to stronger visual activations. Such stronger visual responses for low contrast words were observed before in a PET study^13^. Possibly due to the lower temporal resolution, the initially much stronger responses towards high contrast words were missed and the late but rather sustained activity of low contrast words affected the PET signal more strongly^13^.

**Supplementary References**

1. Moors, A. *et al.* Norms of valence, arousal, dominance, and age of acquisition for 4,300 Dutch words. *Behav. Res. Methods* **45,** 169–177 (2013).

2. Bradley, M. M. & Lang, P. J. Measuring emotion: the Self-Assessment Manikin and the Semantic Differential. *J Behav Ther Exp Psychiatry* **25,** 49–59. (1994).

3. Litvak, V. *et al.* EEG and MEG data analysis in SPM8. *Comput. Intell. Neurosci.* **2011,** 1–32 (2011).

4. Litvak, V. & Friston, K. Electromagnetic source reconstruction for group studies. *Neuroimage* **42,** 1490–8. doi: 10.1016/j.neuroimage.2008.06.022. (2008).

5. Campo, P. *et al.* Anterobasal temporal lobe lesions alter recurrent functional connectivity within the ventral pathway during naming. *J. Neurosci.* **33,** 12679–88 (2013).

6. Shattuck, D. W. *et al.* Construction of a 3D probabilistic atlas of human cortical structures. *NeuroImage* **39,** 1064–1080 (2008).

7. Keuper, K. *et al.* Early prefrontal brain responses to the Hedonic quality of emotional words--a simultaneous EEG and MEG study. *PloS One* **8,** e70788 (2013).

8. Keuper, K. *et al.* How ‘love’ and ‘hate’ differ from ‘sleep’: Using combined electro/magnetoencephalographic data to reveal the sources of early cortical responses to emotional words. *Hum. Brain Mapp.* **35,** 875–88 (2014).

9. Scott, G. G., O’Donnell, P. J., Leuthold, H. & Sereno, S. C. Early emotion word processing: evidence from event-related potentials. *Biol. Psychol.* **80,** 95–104. doi: 10.1016/j.biopsycho.2008.03.010. Epub 2008 Mar 22. (2009).

10. Bayer, M. & Schacht, A. Event-related brain responses to emotional words, pictures, and faces–a cross-domain comparison. *Front. Psychol.* **5,** 1106 (2014).

11. Kissler, J., Herbert, C., Winkler, I. & Junghöfer, M. Emotion and attention in visual word processing - An ERP study. *Biol. Psychol.* **80,** 75–83 (2009).

12. Schacht, A. & Sommer, W. Emotions in word and face processing: Early and late cortical responses. *Brain Cogn.* **69,** 538–550 (2009).

13. Mechelli, A., Humphreys, G. W., Mayall, K., Olson, A. & Price, C. J. Differential effects of word length and visual contrast in the fusiform and lingual gyri during. *Proc. R. Soc. Lond. B Biol. Sci.* **267,** 1909–1913 (2000).
